# Proteomic analysis of the regenerating liver following 2/3 partial hepatectomy in rats

**DOI:** 10.1186/0717-6287-47-59

**Published:** 2014-11-19

**Authors:** Xiao-Guang Chen, Cun-Shuan Xu

**Affiliations:** Animal Science and Technology School, Henan University of Science and Technology, Luoyang, 471003 Henan Province China; Key Laboratory for Cell Differentiation Regulation, Henan Normal University, Xinxiang, 453007 Henan Province China

**Keywords:** Partial hepatectomy, Rat liver regeneration, Proteomic, MALDI TOF/TOF MS

## Abstract

**Background:**

Liver regeneration (LR) after 2/3 partial hepatectomy (PH) is one of the most studied models of cell, organ, and tissue regeneration. Although the transcriptional profile analysis of regenerating liver has been carried out by many reserachers, the dynamic protein expression profile during LR has been rarely reported up to date. Therefore, this study aims to detect the global proteomic profile of the regenerating rat liver following 2/3 hepatectomy, thereby gaining some insights into hepatic regeneration mechanism.

**Results:**

Protein samples extracted from the sham-operated and the regenerating rat livers at 6, 12, 24, 72, 120 and 168 h after PH were separated by IEF/SDS-PAGE and then analyzed by MALDI-TOF/TOF mass spectrometry. Compared to sham-operated groups, there were totally 220 differentially expressed proteins (including 156 up-regulated, 62 down-regulated, and 2 up/down-regulated ones) identified in the regenerating rat livers, and most of them have not been previously related to liver regeneration. According to the expression pattern analysis combined with gene functional analysis, it showed that lipid and carbohydrate metabolism were enhanced at the early phase of LR and continue throughout the regeneration process. Ingenuity Pathway Analysis indicated that YWHAE protein (one of members of the 14-3-3 protein family) was located at the center of pathway networks at all the timepoints after 2/3 hepatectomy under our experimental conditions, maybe suggesting a central role of this protein in regulating liver regeneration. Additionally, we also revealed the role of Cdc42 (cell division cycle 42) in the termination of LR.

**Conclusions:**

For the first time, our proteomic analysis suggested an important role of YWHAE and pathway mediated by this protein in liver regeneration, which might be helpful in expanding our understanding of LR amd unraveling the mechanisms of LR.

**Electronic supplementary material:**

The online version of this article (doi:10.1186/0717-6287-47-59) contains supplementary material, which is available to authorized users.

## Background

The liver is one of the vital organs of the body with mutiple important responsibilities, including metabolism, maintenance of water balance, bile acid production and excretion, detoxification, immune response and so on [1]. In addition, the liver becomes distinguished from other organs mainly by its amazing regenerative ability, which is primarily attributable to the quick reentry of highly differentiated quiescent hepatic cells into the cell cycle in response to liver injury induced by surgical resection (e.g., surgerical resection, pathogenic or chemical factors [2]. Among various liver injury models, partial hepatectomy (PH), resulting in the loss of approximately 70% of the liver volume, is now widely utilized for studying liver regeneration in experimental animals. According to previous reports, many cytokines (e.g., TNF-α, IL-6) are upregulated within 0-6 h after 70% liver resection. DNA synthesis begins at 12-16 h following PH, peaking around at 24-48 h, which results in a steep increase of liver mass at 72 h after PH, followed by the rough restoration of liver mass to normal around at 168 h after PH [3]. In general, rat liver regeneration lasts about 7 days, during which involves many biological events including cell activation, cell differentiation, proliferation and its regulation, redifferentiation, reestablishment of liver structure and function as well, inferring that the mechanism underlying this process is highly complex [4]. Therefore, in order to understand clarify the regenerative process of the liver, it is very essential to clarify the overall and systematically molecular basis of this stringently regulated process.

So far, the high-throughput analysis technologies, such as genechip, serial analysis of gene expression (SAGE), have been extensively used by researchers to identify transcriptome profiles of the regenerating livers, and obtained the valuable data for understanding mechanism of LR. Especially in 2009, Wang et al. analyzed the gene expression profiling of regenerating rat livers at different recovery time points after PH, finding that there are totally 1004 known genes and 857 unknown genes associated with LR [5]. However, the data from microarray analysis is too limited to quantitatively analyze protein levels, or even to reflect the final biological effect of genes. So, despite that the gene expression pattern has come under intense scrutiny, a differential proteomic study could better help to elucidate how the process is triggered and regulated.

Recently, the emergence of comparative proteomics technologies, such as two-dimensional electrophoresis (2-DE), high performance liquid chromatography (HPLC), mass spectrometry (MS) and mass fingerprinting, provides a promising approach for elucidating the mechanism of LR [6]. As a protein separation technique, 2-DE has been widely employed in separating and quantifying proteins that are differentially expressed in proteomics research. Depending on this method, the proteins whose levels were significantly changed under a defined physiological condition can be screened out and identified. For instance, Strey and his coworkers applied two-dimensional gel electrophoresis approach to measure protein expression changes in mouse livers at 6 h and 12 h after PH, and identified twelve up-regulated (at least 2-fold) proteins related to signaling and metabolic pathways [7]. Nevertheless, this technology has some striking limitations such as low load ability, poor separation of hydrophobic, acidic and alkaline proteins. And HPLC or MS techniques overcome the shortcomings of 2-DE, and suit differential proteomics study much better. Also, other techniques such as multi-dimension chromatogram, protein chips have been utilized to complement or substitute this conventional method. Presently, the method of 2-DE separation combined with MS identification is now in common use in this research field. For example, He *et al.* introduced this method to detect protein expression profiles in rat regenerating livers at 1 hour after PH, and identified a total of 24 differentially expressed proteins. In addition, He’s research team applied 2-DE in combination with MALDI-QTOF-MS compared protein expression patterns between sham-operation group and 7-hour hepatectomized group to identify proteins whose expression may be altered after PH, and discovered 29 differentially expressed proteins [8]. In study by Hsieh et al., they used iTRAQ-coupled LC-MS/MS technique to perform a comparative analysis of protein expression profiling in mouse liver regenerating for 24, 48 and 72 h after PH, and found a total of 270 time-dependently differentially expressed proteins during the regenerative process [9]. Up to date, these technologies are often employed to study the early event occurring in the first 24 hours after surgery, failing to comprehensively display the dynamical changes in protein expressions during LR. For this reason, in this study, we assessed the temporal expression of proteins from the regenerating liver recovering 6, 12, 24, 72, 120 and 168 h following 2/3 PH in rats with matrix-assisted laser desorption/ionization time-of-flight mass spectrometry (MALDI TOF/TOF MS), thus establishing proteome altas of the normal liver and regenerating liver, which might lay the foundation for further screening out the key factors and cell markers associated with LR.

## Results

### Changes in liver-to-body weight ratio duirng rat liver regeneration

**Figure 1:**
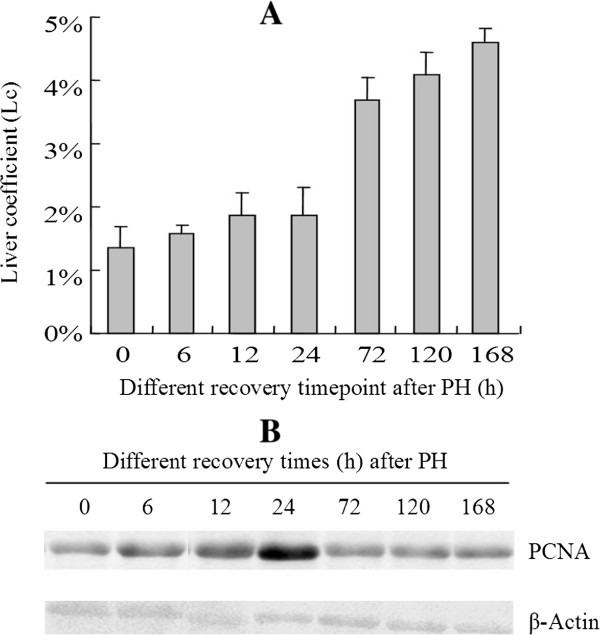
**The liver coefficient changes during rat liver regeneration (A) and Western blot of PCNA (B) in whole-liver extracts prepared from the control and the regenerating liver tissue samples at six different timepoints after PH.** β-Actin was used as a loading control.

In this study, rat body weight (g) and regenerating liver weigh (g) at each timepoint were weighed, and the ratio of liver weigh to body weight was defined as liver coefficient (Lc) . According to the calculation results, the liver coefficients at 0, 6, 12, 24, 72, 120, and 168h after PH in rats were 1.35%, 1.58%, 1.86%, 1.86%, 3.69%, 4.08% and 4.61%, respectively (Figure 1A), and the time-dependent increase in liver coefficients demonstrated the sucessesful liver regeneration after PH.

To testify whether the livers reliably regenerated after PH at the molecular level, we chose PCNA (an auxiliary protein of DNA polymerase delta that accumulates in the late G1 and early S-phase and whose level correlates with cellular prolifeative activity) to perform a western-bloting assay. As shown in Figure 1B, compared to the control (0 h), PCNA levels started to increase at 6 hours and peaked at 24 hours after PH, followed by a gradual reduction, indicating an enhanced cell proliferation during 12-24 hours, which is consistent with previous reports by other researchers.

### Comparison of structural changes in normal and regenerating livers in rats

**Figure 2:**
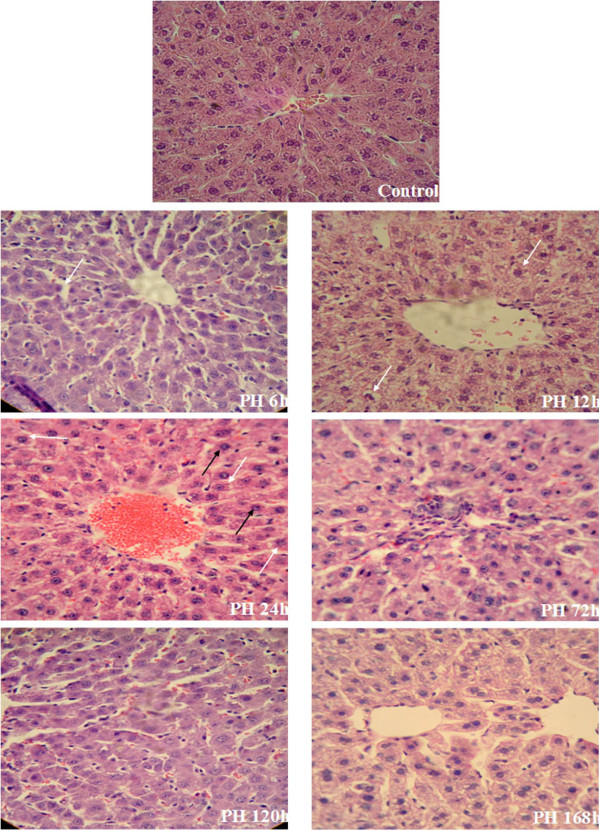
**Histological examination of regenerating livers at different recovery time points post 2/3 PH in rats.** Subfigures respectively indicate the liver histology slide of 2/3 PH group 0 h, 6 h, 12 h, 24 h, 72 h, 120 h, 168 h after operation (HE staining 400×). White arrows: liver cell necrosis; Black arrows: the increased hepatocyte cell size.

HE staining indicated the normal hepatic architecture with typical hepatic lobule and hepatic sinusoid, radial liver cell cord and uniform distribution of the cells in the control samples. By contrast, pathological changes were seen in the 2/3 hepatectomized livers: 6 h to 24 h post PH, there was more and more serious liver necrosis whose feature was that a number of hepatocytes showed a marked increase in nuclear size, accompanied with vesicular bodies and prominent nucleoli caryocinesia; 72 h to 120 h, the degeneration of hepatic cells and destruction of hepatic architecture were alleviated, but there were still many hepatocytes with enlarged nuclei indicative of cell division. Until 168 h after PH, the histological structure of regenerating liver closely resembled the normal liver tissue (Figure 2).

### Differentially expressed proteins in regenerating livers after 2/3 PH in rats

**Figure 3:**
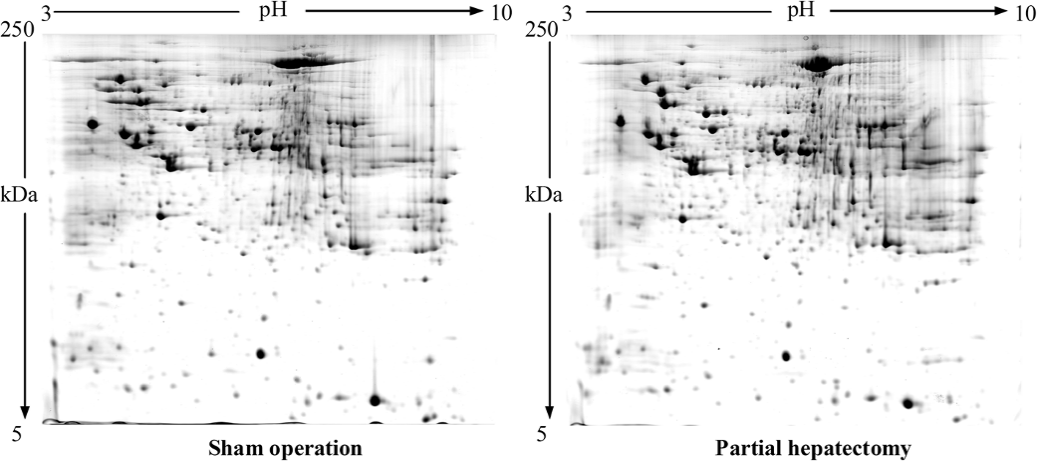
**Representative 2-DE maps of rat liver tissues corresponding to sham operation group (left) and PH group (right) at 24 hour after PH.** Protein spots were stained with colloidal Coomassie stain. The 2-DE was repeated at least three times for each group.

We collected regenerating liver samples from 6h through 168h post 2/3 PH, and analyzed protein expression profiling at six different timepoints after surgery.To save space, we only showed the 2-DE maps of SO group and PH group at 24 hour because this is the time point when DNA and protein synthesis are most active (Figure 3). Among 2546 and 2554 differentially expressed proteins identified respectively in sham-operated groups and 2/3 hepatecyomized groups, 220 showed statistically significant differences (P < 0.05) in levels between PH group and SO group. These proteins were also called LR-related proteins whose volume changes in sham-operated rats and 2/3 hepatectomized rats were detailedly shown in Additional file 1: Table S1 of the Supporting Information. They were categorized into six groups based on expression changes: group 1, 125 proteins were up-regulated after 2/3 PH; group 2, 26 newly induced proteins were detected only in PH group but not in SO group; group 3, 5 proteins were down-regulated only in SO sample; group 4, 28 proteins were down-regulated after 2/3 PH; group 5, 34 proteins were below detection limit in PH group (detected only in SO sample but not in 2/3 PH sample); and group 6, 2 proteins were up-regulated at early phase but down-regulated at late phase during LR. In a general sense, proteins in group 1, 2 and 3 (totally 156) were viewed as up-regulated proteins during LR, and ones in group 4 and 5 (totally 62) as down-regulated proteins, and the remaining two in group 6 as up/down-regulated proteins.

### Protein functional categorization

The 220 LR-related proteins identified in this study were divided into eight groups according to their biological functions (shown in Additional file 1: Table S1): (1) carbohydrate, lipid, protein and energy metabolism, involving 63 proteins; (2) amino acid and nucleic acid metabolism, involving 32 proteins; (3) biotransformation, involving 9 proteins; (4) cell proliferation-ralated protein, involving 13 proteins; (5) cell differentiation and development-related proteins, involving 55 proteins; (6) signal transmission, involving 23 proteins; (7) inflammatory factors and related proteins, involving 12 proteins; and (8) others, involving 13 proteins which were mainly up-regulated in LR and were hard to categorize into special biological activity.

Sixty three of the identified proteins were involved in carbohydrate, lipid, protein and energy metabolism. Of these proteins, 43 proteins were upregulated including 9 carbohydrate metabolism-related (mainly involved in catabolic process, i.e., G6PD), 13 lipid metabolism-related (mainly lipid degradation proteins, i.e., ACADVL, HADH), 17 protein metabolism-related, and 4 energy metabolism-related proteins (i.e., COX5A, UQCRC1, ATP6V1B2, ATP5B); 20 proteins were downregulated including 4 carbohydrate metabolism-related, 8 lipid metabolism-related, 7 protein-metabolism and 1 energy metabolism-related proteins. Thirty-two proteins were found to be functionally related to amino acid and nucleic acid metabolism, 18 proteins of which were upregulated in PH rats at early phase after operation, such as GOT1, ARG1; the remaing 14 were decreased in expression levels in 2/3 hepatectomized rats versus sham-operated rats, such as GLUL, RNASEH1.

Thirteen proteins, increased primarily in the middle phase of rat LR, were functionally related to cell proliferation. These proteins were involved in various events occurring in the cell cycle, such as chromatid separation (i.e., TOP2a), G1/S transition (i.e., PSMC4), and the regulation of cell cycle progression (i.e., CDC42).

Fifty-five proteins were functionally associated with cell differentiation and development, such as extracellular matrix organization-involved components (i.e., VWA1), cytoskeleton organization-related factors (i.e., NUP35) and so on. Out of these proteins, 43 were increased in hepatectomized rats, and the other 12 were decreased.

Twelve proteins were identified as inflammatory factors and the functionaly related proteins. Eight of them were upregulated after PH in rats, and other four were down-regulated. These proteins mainly play roles in antigen processing and presentation (i.e., PSMA6), macrophage chemotaxis (i.e., EDN2), and histocompatibility antigens (i.e., RT1-B) as well.

Twenty-three proteins were identified to be responsible for signal transduction, the majority of which (involving 18 proteins) were up-regulated during the regeneration process. They participate in various signal pathways, such as Ras signaling pathway (i.e., RASA2), cAMP pathway (i.e., CAP1), epidermal growth factor receptor signaling pathway (i.e., PDGFRB), GABA signaling pathway (i.e., GABRB2), Wnt receptor signaling pathway (i.e., AXIN2), Rho signal transduction (i.e., ARHGDIA), and insulin receptor signaling pathway (i.e., AKT2). These signaling pathways modulate multiple biological processes as mentioned above, such as cellular metabolism, cell proliferation, cell differentiation and inflammatory response *etc*.

### Pathways regulating rat liver regeneration after 2/3 PH

**Figure 4:**
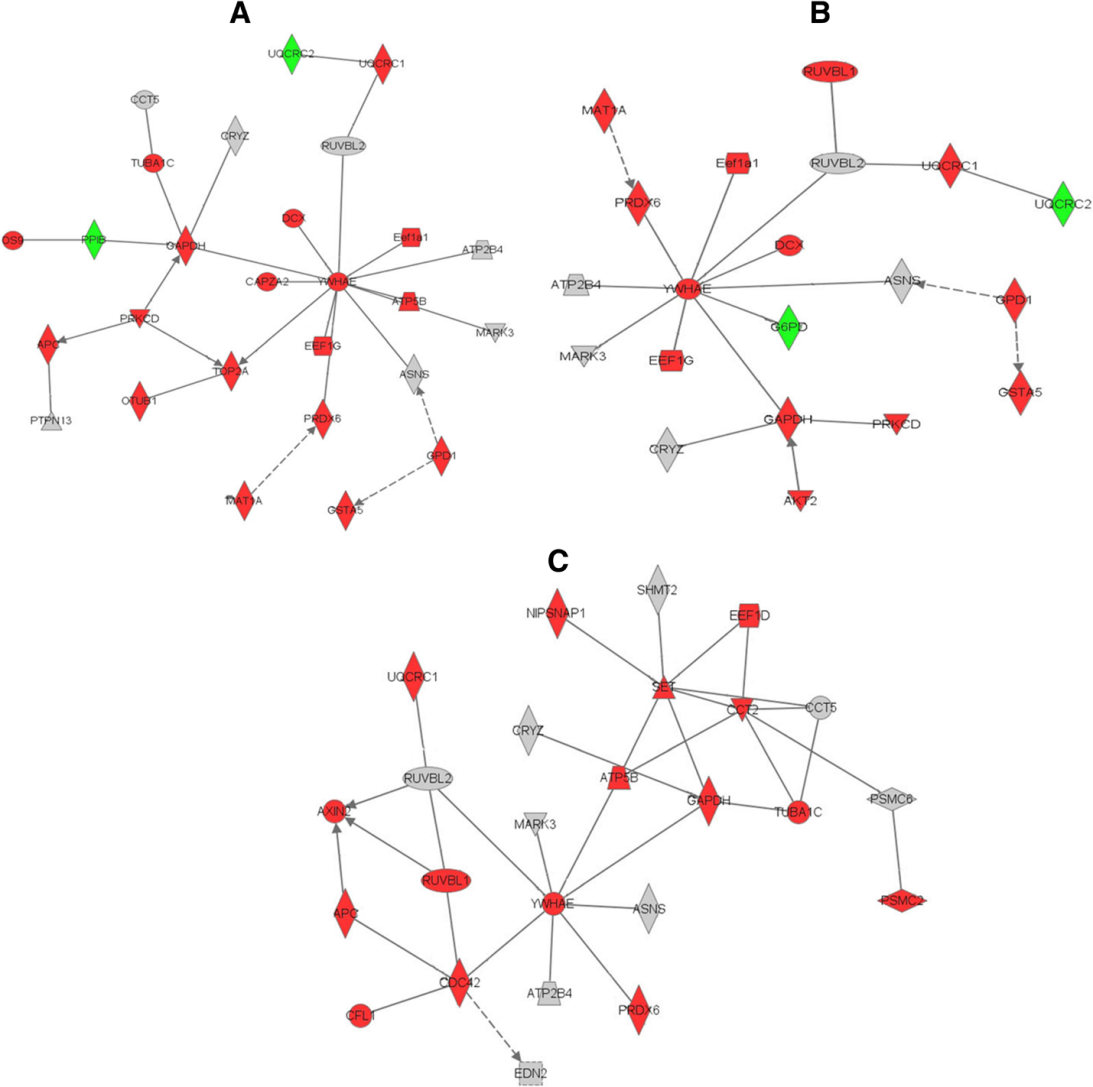
**Pathway analysis results that differentially expressed proteins at three time points including 6 h (panel A), 24 h (panel B) and 168 h (panel C) after 2/3 PH in rats can be sorted into different biological pathways.** There are 18, 19, 14 root nodes in the sub-networks from 6-, 72- and 168-hour regenerating livers, respectively. The root nodes in above three subnetworks are all connected to YWHAE directly or indirectly. Red and green were identified as upregulated and downregulated proteins, respectively. Other gray denoted the proteins that were down-regulated only in SO sample or detected only in SO sample. Lines connecting the molecules indicate molecular relationships. Real lines indicate direct interactions and dashed lines indicate indirect interactions.

All the differentially expressed proteins at each time point after PH, that’s 125 proteins at 6 h, 143 proteins at 12 h, 121 proteins at 24 h, 124 proteins at 72 h, 119 proteins at 120 h, and 131 proteins at 168 h, were respectively subjected to pathway analysis using Ingenuity Pathway Analysis 9.0 software. For audience’s convenience, we only presented the networks constructed by differentially expressed proteins at the representative time points during LR (e.g., 6 h within early phase, 72 h within middle phase and 168 h within terminal phase), as displayed in Figure 4. The networks from six recovery timepoints was attached in the Additional file 2: Figure S1.

As Additional file 2: Figure S1 indicated, the differentially expressed proteins of rat regenerating livers from each timepoint after 2/3 PH were connected to each other in one way or another to construct a network distinct from one another. Despite the great difference from each other, the common point between these networks was that 19 of 125, 24 of 143, 14 of 121, 19 of 124, 18 of 119, 14 of 131 proteins at six time points were all clustered into YWHAE protein-mediated pathway. Obviously, YWHAE protein was placed at the center of all the networks and modulated the diverse biological activities, such as cell division, apoptosis, cell differentiation, cell development and so on. According to pathway analysis, at the late phase of LR, besides YWHAE protein, CDC42 is considered as another important factor involved in the events occuring the termination of LR.

### Validation by Western blot analysis

**Figure 5:**
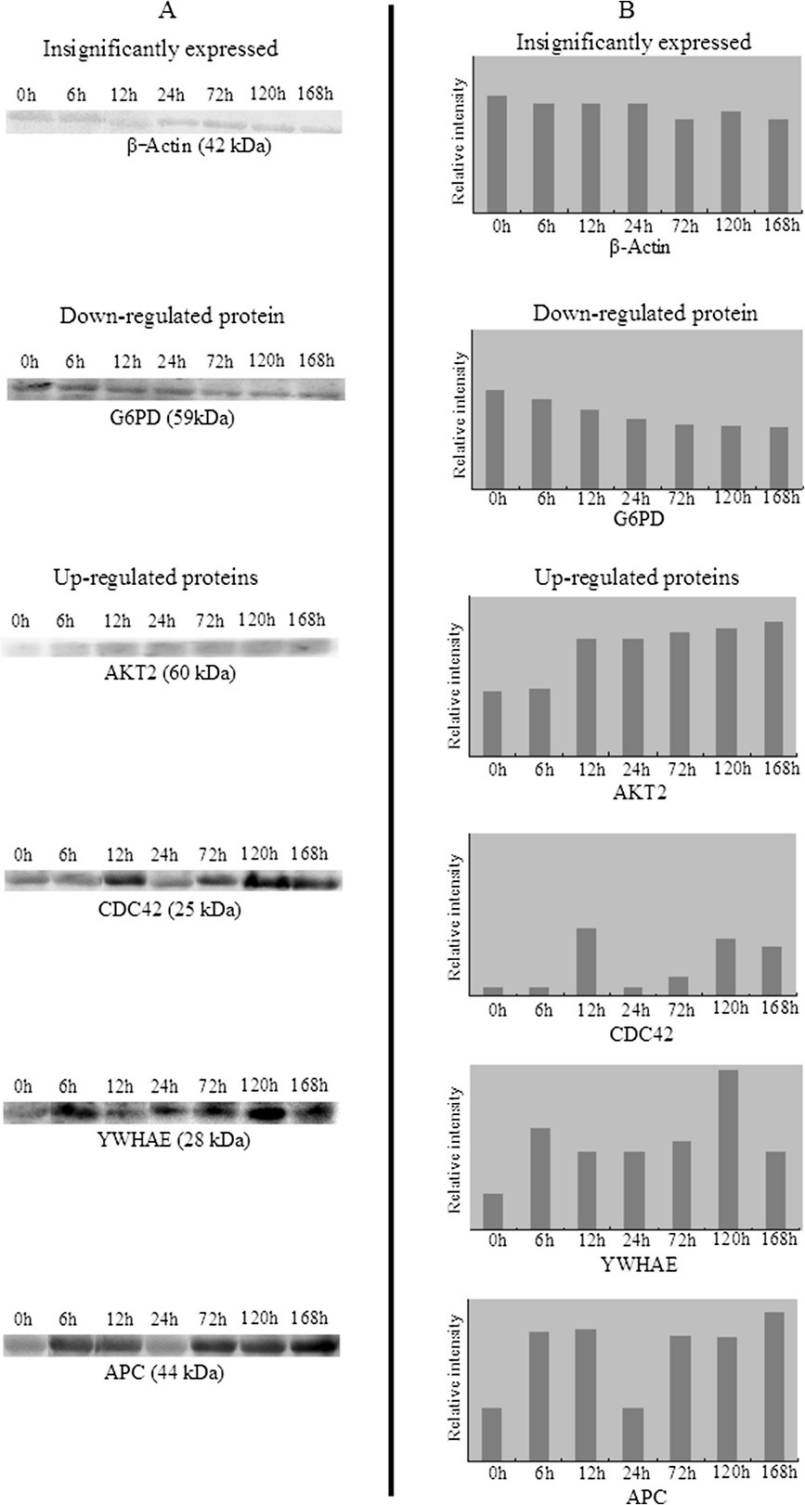
**Western blot analysis validating abundance alterations of β-Actin, G6PD, AKT2, CDC42, YWHAE and APC which was seen by 2-DE in control group (0 h) and PHx group (6, 12, 24, 72, 120, 168 h after surgery). Panel A** showed the Western blot results for Actin, PLCG2, CDC42, AKT2, PDGFRB and APC. Actin was used as a control. **Panel B** showed the gray value of Western blot bands.

To testify the reliability of identification of differentially expressed proteins, six differential proteins in our experiments, including β-Actin, G6PD, AKT2, CDC42, YWHAE and APC, were picked out for Western blotting using available specific antibodies. Figure 5A showed the Western blot results of the six proteins. Figure 5B showed the gray values of Western blot bands obtained with BandScan 5.0 software. The results showed that G6PD was down-regulated in the PH group compared to the sham operation group, while AKT2, CDC42, YWHAE and APC were obviously up-regulated in the PH grou, which were generally consistent with the results of 2-DE experiments.

## Discussion

Studies have shown that, in certain range, the regenerative response is directly proportional to the mass of liver resected ([10] Liver regeneration. Michalopoulos GK, DeFrances MC. Science. 1997;276(5309):60-6]). Usually, the removal of approximately 75% of liver volume was tolerable for normal rodents, but the resection of more than 75% of liver mass often leads to liver failure and death. Accordingly, to induce maximal hepatic regeneration meanwhile maintain sufficient hepatic functionality meeting the essential body metabolic demand, 70% (or 2/3) partial hepatectomy now has been become the most widely used model to study the mechanism of liver regeneration. Using this animal model, a body of evidence has shown that many factors (e.g., HGF, NFκB, TNF and IL6) participate in the initiation of LR [11]. Also, based on this model, many genomic and proteomic studies have been carried out to map the complex dynamic expression changes of genes/proteins at the early phase of LR. However, the dynamical changes of protein expressions during the whole regeneration process have been rarely reported. So, the present study attempted to detect the overall dynamic proteome changes following PH in rats, gaining some insights into the nature of LR.

We categorized all the 220 liver regeneration-associated proteins into six groups. In group 1, 125 proteins were up-regulated post PH in contrast to SO group. In addition, in group 3, 5 proteins were down-regulated only in sham-operated rat livers but obviously unchanged in the hepatectomized liver, thus also sorted in the “up-regulation” pattern. Among these 130 proteins, 25 have been reported to be associated with liver regeneration according to the previous studies (Additional file 1: Table S1), and mainly play the role in lipid metabolism (ACADM, HMGCS2 and PRDX6), carbohydrate and energy metabolism (COX5a, GPD1, PDHA1 and UGP2), amino acid and nucleic acid metabolism (ALDH6a1, ARG1, CPS1, GOT1, HPD and UROD), differentiation and development (EEF1A1, EIF5A, GMFB, KRT6A, NDUFS2 and RGN), signal transmission (ARHGDIA and YWHAE), or inflammatory response (GSTM2 and PDIA3). In terms of the expression pattern, a substantial portion of these proteins began to be dramatically up-regulated at early phase. Particularily, the up-expression of metabolism-related proteins last throughout the entire regenerative process, consistent with Guo’s report that lipid and carbohydrate metabolism were significantly enhanced, which not only compensates metabolic homeostasis, but also can function as driver of the complex cascade of gene activation required for cell proliferation [8]. In addition, some studies suggested that, immediately after PH, inflammatory factors STAT3, TNFα and IL6 were activated to regulate the expression of a large number of genes involved in inflammation, acute phase response and cell proliferation. For example, Taub pointed out that, although an obvious inflammatory response was not seen in liver parenchyma at the initial phase of liver regeneration, there occurred the elevated levels of acute-phase proteins and the release of cytokines responsible for the regulation of inflammation [12]. In general, our findings were in accordance with his statement. However, the up-regulated inflammatory factors in our study were not STAT3, TNFα and IL-6, but GSTM2 and PDIA3 identified as LR-related proteins by other studies [12]. The reason for the discrepancy between ours and others might be attributable to the different experimental conditions, different example batches, different operational methods and so on. It is worthily emphasized that our study detected the an up-regulation (a peak of 2.2-fold at 120 h) of inflammation-related protein GSTM2 which was found to be down-regulated in the Cao’s study using 50% PH mouse model [13]. The difference between results from the two studies may be due to different experimental animals and operational procedures.

Partial hepatic resection in rats induced a number of mediators including inflammatory cytokines, growth factors and various hormones which can activate a complex network of signal transduction that promotes hepatic regeneration. As described previously, the signaling pathway mediated by IL6, TNFα and STAT3 is essential for triggering live regeneration. However, the expressions of these cytokines didn’t show the significant changes in this present study. Instead, signal transmission-related YWHAE, a LR-associated protein identified by Li *et al.*[14], was dramatically up-regulated by 7.6-fold at 6h post PH. According to the Ingenuity Pathway Analysis conducted by us, the networks from six timepoints after 2/3 hepatectomy all centered around YWHAE protein. Although Li *et al.* provided the evidence that YWHAE was related to regeneration process [14], before that, nobody realized its importance to hepatic regeneration. YWHAE belongs to the 14-3-3 family of proteins that can signal by binding to phosphoserine-containing proteins and plays a role in a wide variety of cellular functions. Besides functioning in cell division and regulation of insulin sensitivity, its other specific functions need to be further identified. And our study suggested the significance of this protein in LR, which might be the first observation of a new pathway critical to the regenerative process. Besides the newly-identified YWHAE-mediated pathway, cell division control protein 42 (CDC42) was considered as another important factor that directly or indirectly connected to LR termination-involved proteins including APC, CFL1, RUVBL1 and so on. Our data showed that the expression of this protein was 2-fold up-regulated at the termination of LR (including 120 h and 168 h). As a small GTPase of Rho-subfamily, CDC42 plays important roles in diverse cellular functions such as cell morphology, migration and cell cycle. Therefore, it could be concluded that CDC42 was key to the termination of LR. Consistent with this, some protein involved in the end of liver regeneration, such as APC, CFL1, also showed the two-fold up-regulation at the same period, which reinforced the above conclusion drawn by us. Whereas, Yuan and his colleagues used a mouse model with liver-specific knockout of CDC42 and studied the function of CDC42 in hepatic regeneration after PH. Their results showed that the loss of CDC42 led to a significant delay of liver recovery after PH, suggesting the important role of CDC42 in regulating proliferative response during LR [15]. By contrast, our study observed another action of CDC42 in hepatic recovery.

Among the remaining 100 up-regulated proteins, of particular note are GAPDH (up-regulation during the whole LR with a peak of 26.4-fold), the enzyme catalyzing an important energy-yielding step in carbohydrate metabolism. This protein was sorted into the YWHAE-mediated pathway according to our pathway analysis (Additional file 2: Figure S1). The elevated mRNA and protein levels of GAPDH at regenerating rat liver after 70% PH has been reported, which might result directly from the stimulating effect of insulin [16, 17]. As stated previously, the sensitivity of body tissues to insulin is stimulated by YWHAE-mediated pathway. Thus, the significant up-regulation of GAPDH in our study might possibly reflect the up-regulation of YWHAE and the enhancement of YWHAE-mediated pathway. Secondly, another emphasized protein was TUBA1C that was significantly up-expressed during the whole regeneration process, with a peak of 19.3 folds at 24 h following PH. This protein is one of subunits of tubulin which acts as the main component of the microtubule cytoskeleton and play a crucial role in regulating the mitotic spindle during cell division. In this study, alpha-tubulin protein level was detected to have a 1.3-23.0 fold increase in rat regenerating liver, which was agreeable with the Matos’s observations that the level of this cytoskeletal components in liver tissues was strikingly increased in patients with hepatocellular carcinoma [18]. According to above description, to a certain extent, there existed similarity in some respects between liver regeneration and liver cancer.

In group 2, 26 proteins were detected only in partially-hepatectomized rats instead of the sham-operation group, so called “newly-induced proteins”. The failure to detect those proteins in SO group could be due either to true absence of their expressions or to their very low quantity below the detection limit of our detection. Nearly half (12 out of 26) of the “newly-induced proteins” in response to 2/3 hepatectomy were related to cell differentiation and development. In recent decades, there has been an increasing interest in the relationship between liver regeneration and nervous system. Previous researches observed the axonal growth around the portal area in rat liver regeneration model [19]. Similarily, our results showed that, among the 12 cell differentiation and development-involved proteins, neuronal migration protein doublecortin (DCX), neuron-specific calcium-binding protein hippocalcin (HPCA) and heart- and neural crest derivatives-expressed protein 2 (HAND2) have been recognized as the important factors in nervous system development. DCX directed neuronal migration by regulating microtubule organization [20]. HPCA and HAND2 play the important roles in neural regeneration of nervous system [21]. Overall, all of them may be responsible for neuron growth in remnant liver after PH. Our finding was similar to the one reported by Sun et al. [19] where the new synthesis of neuromodulin and GMFB had respectively a 2.6- and 3.1- fold increase at 1h after PH, in despite of the difference between the differential proteins identified by us and that by Sun. In group 4 and group 5, proteins are down-regulated or below detection limit after treated with 2/3 PH, respectively. Among these down-regulated proteins, carbohydrate, lipid and energy metabolism-involved enzymes were particularly noteworthy.

Carbohydrate metabolism-involved enzymes including G6PD, CTSA, PGLS, PYGL, TKT and UQCRC2 showed the down-regulation, ranging from 1.2 to 7.7 fold decrease. Lipid metabolism enzymes or related proteins including ACAA2, ALOX15B, ECI1, FABP7, FABP9, IAH1 and LIPK were also decreased in expression level with a range from 1.1 to 5.8 fold reduction. Two classes of above proteins were mainly responsible for glucose catabolism or fatty acid oxidation. So, their down-regulation might lead to a reduced glucose and fatty acid degradation process. But combining with the expression profiles of the related proteins in group 1, the weakened metabolic process caused by the down-regulation of these metabolism related-proteins was not enough to offset the enhancement of these metabolism activities induced by the up-regulation of a majority of glucose and fatty acid degradation-involved proteins. It has been widely accepted that energy production in the body mainly derives from the catabolism of carbohydrate and fatty acid. Therefore, according to our analysis it could be proposed that, to some extent, cellular ATP production would be increased. However, Cao *et al.* showed that some key enzymes involved in citric acid cycle and electron transport chain were down-regulated or below detection limit, implying that ATP production would be hampered [13]. Crumm *et al.* also reported that ATP level in the remaining livers after 30% PH was remarkably decreased and remained significantly lower than SO samples between 24-48 h post PH [22]. Clearly, our conclusion was quite opposite to that of Cao *et al.* and Crumm *et al.*, and the reason remains unclear and needs to be answered by means of further studies.

## Conclusions

According to expression and functional analysis of differentially expressed proteins, the metabolic processes of lipid and carbohydrate were significantly promoted at early phase of LR. Ingenuity Pathway Analysis suggested that YWHAE protein was located mainly in the center of the networks at all the six recovery timepoints after 2/3 hepatectomy, considering this protein as one of the master factors regulating liver regeneration under our experimental conditions. And its exact role in liver regeneration needs to be further explored using other methods, such as gene addition, RNA interference and so on. Additionally, our study also showed the implication of CDC42 in the termination of LR through mediating the specific pathway. All in all, our work may be helpful in expanding our understanding of liver regeneration and hepatic response in acute liver diseases, and it might ultimately lead to the emergence of novel therapeutic strategies to accelerate liver regeneration after hepatic injury.

## Methods

### Preparation of animal models

114 adult healthy Sprague-Dawley (SD) rats, weighing 200 ± 20 g each, were obtained from the Experimental Animal House at Henan Normal University, and randomly divided into 19 groups (six in each group): one control group (0-h group), nine sham-operation (SO) groups and nine 2/3 PH groups. For the control group, rat liver tissues were totally resected. For the 2/3 PH groups, the rats were subjected to an operation removing approximately 70% of the liver as described by Higgins and Anderson [23]. The rats from nine PH groups were sacrificed at 6, 12, 24, 72, 120 and 168 hours after surgery, respectively. Of the 3 liver tissue samples resected from each rat, one was fixed with 4% paraformaldehyde and embedded in paraffin for histological examination using HE staining, and the other two samples were stored at -80°C until samples from all time points are ready to be analyzed simultaneously. For SO groups, the animals was subjected to the same operation procedure as rats in PH groups but without liver removal. During the course of this experiment, animals were housed in the standard facilities with a 12-hour day/night cycle (8:00-20:00 each day) and were given free access to food and water. Animal protocols strictly follow the Animal Protection Regulations in China.

### Protein sample preparation

The resected liver tissues was washed in pre-chilled PBS and then cut into pieces. Six samples were combined for each group. The samples were grinded into a homogenous mixture with 2D lysis buffer (7 M urea, 2 M thiourea, 4% CHAPS, 18 mM dithiothreitol [DTT], 0.5% IPG buffer), then centrifuged at 20,000 × g for 45 min at 4°C. The 2-D Clean-Up Kit (GE Healthcare, USA) was used for protein purification and the 2-D Quant Kit (GE Healthcare, USA) for protein quantification.

### Two-dimensional gel electrophoresis (2-DE) and image analysis

IPG strips (24 cm, pH 3-10,) were equilibrated overnight in presence of 750 μg protein samples from each group in 450 μl rehydration solution (8 M urea, 4% CHAPS, 1 mmol/L PMSF, 20 mM DTT, and 0.5% IPG buffer). Isoelectric focusing was performed on the Ettan IPGphor III (GE Healthcare, USA) according to the following program: 30 V for 6 h, 40 V for 7 h, 100 V for 1 h, 250 v for 2 h, 500 V for 2 h, 1000 V for 3 h, 10000 V gradient for 3 h, and 10000 V for 12 h. The samples were then reduced by incubating the strips in SDS-equilibrium buffer (6 M urea, 75 mM Tris-HCl pH 8.8, 29.3% glycerol, 2% SDS, 0.002% bromophenol blue) +1% w/v DTT for 15 min, and alkylated using SDS-equilibration buffer +2.5% w/v iodoacetamide for another 15 min. The strips were then placed onto 12.5% polyacrylamide gels for electrophoresis using Amersham Ettan DALT Six system. Once completed, the gels were fixed overnight in 10% acetic acid and 30% ethanol.

The 2-DE gels were developed by coomassie brilliant blue (G-250) staining according to the method of Candiano *et al.*[24] and scanned with the Image Scanner III (GE Healthcare, USA). Protein profiles were evaluated using the ImageMaster 2D Platinum 7.0 (GE Healthcare, USA). Briefly, protein spots were firstly detected automatically and then refined manually, followed by normalization and matching of the gels. For each protein spot, in the gel the ratio of its volume to the sum of all the spots volume were calculated and used for quantitative comparison. Each inter-group comparison of the samples between control group and test group was done on three separate paired gels. the protein spots can be considered a genuine difference in actual protein expression, if meeting the following criterion: show similar qualitative changes in all three paired repeats of a given comparison and the expression change was at least twofold or the p < 0.05.

### Tryptic in-gel digestion

Protein spots were excised from the coomassie brilliant blue stained gels and were in-gel digested following the method of Yu *et al.*[25]. Gel plugs were soaked in Milli-Q water for washing twice (10 min each), then destained with ammonium bicarbonate buffer (25 mM NH_4_HCO_3_): 50% acetonitrile (ACN) (1:1; v/v). Subsequently, the gel plugs were dehydrated by incubating in 100 μl ACN for 10 min and left to dry naturally. Proteins were then digested with 0.01 ug/ul trypsin in 25 mM ammonium bicarbonate for 30 min at 4°C. One μl aliquot was spotted onto a MTP AnchorChip™ 800/384 sample plate with 1 μl matrix solution (4 mg/ml α-cyano-4hydroxycinnamic acid [HCCA] in 0.1% trifluoroacetic acid [TFA] in 70% acetonitrile [ACN]) and was dried at room temperature.

### MALDI-TOF/TOF MS analysis and data processing

Samples were analyzed in the AutoFlex III™ MALDI-TOF/TOF mass spectrometer (Bruker Daltonics, Bremen, Germany). Mass spectra were initially acquired in reflection mode in a mass range of 700 to 4200 m/z. Then, the instrument was switched to MS/MS (TOF/TOF). MS/MS spectra were acquired using collision-induced dissociation (CID) with atmospheric air as the collision gas. All mass spectra were calibrated externally with a peptide mass standard kit (Bruker) and internally with trypsin autolysis peaks. MS and MS/MS spectra from the same spot were merged in a single mgf-file (MASCOT generic format) prior to submission for database searching.

MGF files were submitted to the MASCOT database search program (Matrix Science Ltd., London, UK) for searching against all the databases. The research parameters were following: trypsin with one missed cleavage allowed; fixed and variable modifications were cysteine carbamidomethylation and of methionine oxidation, respectively; peptide tolerance for MS and MS/MS spectra was 100 ppm and 0.25 Da, respectively; signal-to-noise ratio was 30:1 and 20:1, respectively, for MS and MS/MS; at least 3 tryptic peptide fragments were matched to a particular protein.

### Identification of liver regeneration-associated proteins

The relative quantity of one particular protein is presented as the average value of three independent experiments. When comparing each treatment group with a control group, the protein spots with ≥2.0-fold change were regarded as being differentially expressed. In detail, the protein with ≥2-fold higher than the control was considered as up-regulated; the protein with ≥2-fold lower than control, as down-regulated; the protein with 0.5 ~ 2-fold, as non-differentially expressed. At the same time, the difference between protein expression level in PH group and that in SO group was statistically analyzed with a T-test. A p value <0.05 was considered as significant. The differentially expressed proteins whose expressions have significant (P ≤ 0.05) or very significant (P ≤ 0.01) differences between PH group and SO group, are considered as liver regeneration-related proteins.

### SDS-PAGE and Western blotting

Western blotting technique was used to verify the differentially expressed proteins identified in this study. Firstly, Proteins from liver tissues of the control group and PH-treated groups were separately pooled and subjected to 12.5% SDS-PAGE gel electrophoresis according to the method of Laemmli [26]. The separated proteins were transferred onto nitrocellulose membrane (NC) membranes. Subsequently, the membranes were blocked with 5% nonfat milk in PBST containing 0.1% Tween 20 (Sigma) for 1 hour at 37°C and washed three times (5 min each time) with PBST. After blocking, the membranes were incubated in primary antibody overnight at 4°C and washed three times in PBST, followed by the exposure of the membranes to alkaline phosphatase-labeled secondary antibody for 1 hour at 37°C and developing with color liquid in dark for 15-30 min at 37°C. The reaction was stopped with TE buffer. The digital image was obtained by scanning the membrane on the densitometer, and then was subjected to gray value analysis.

### Pathway analysis

Pathway network analysis of the identified proteins differentially expressed at the 6-, 12-, 24-, 72-, 120- and 168-h time points were carried out with the Shortest Paths and the Analyze Network options of Ingenuity Pathway Analysis 9.0 software, a web-delivered application (http://www.Ingenuity.com) that can identify major biological themes during LR and predict the key networks functioning at different phases of regeneration process. The Shortest Paths option can incoporate all given proteins into the shortest possible paths. The network is built dynamically, connecting all the proteins through direct mechanistic interactions on the basis of manually curated publications.

## Electronic supplementary material

Additional file 1: Table S1: Protein expression profiling of both sham-operated groups (highlighted in red) and partial-hepatectomized groups (highlighted in blue) in six clusters. Note: The gray-colored bins represent the ≥2-fold up-regulation; the spot-marked bins represent ≥2-fold down-regulation; the colorless bins represent the insignificant expression. Asterisk denotes those proteins that have been identified to be LR-related in other studies. (XLS 84 KB)

Additional file 2: Figure S1: Pathway analysis results showing that differentially expressed proteins at each time points after 2/3 PH in rats can be sorted into a specific biological pathway. The root nodes in six subnetworks are all connected to YWHAE directly or indirectly. Red and green proteins were identified as differentially upregulated and downregulated, respectively. Other gray denoted the proteins that were down-regulated only in SO sample or detected only in SO sample. Lines connecting the molecules indicate molecular relationships. Real lines indicate direct interactions and dashed lines indicate indirect interactions. (JPEG 192 KB)
