# Supplementary figures and images for: Proteomic analysis of the regenerating liver following 2/3 partial hepatectomy in rats
Source: Biol Res. 2014 Nov 19;47(1):59. doi: 10.1186/0717-6287-47-59 (PMC4335715; doi:10.1186/0717-6287-47-59)

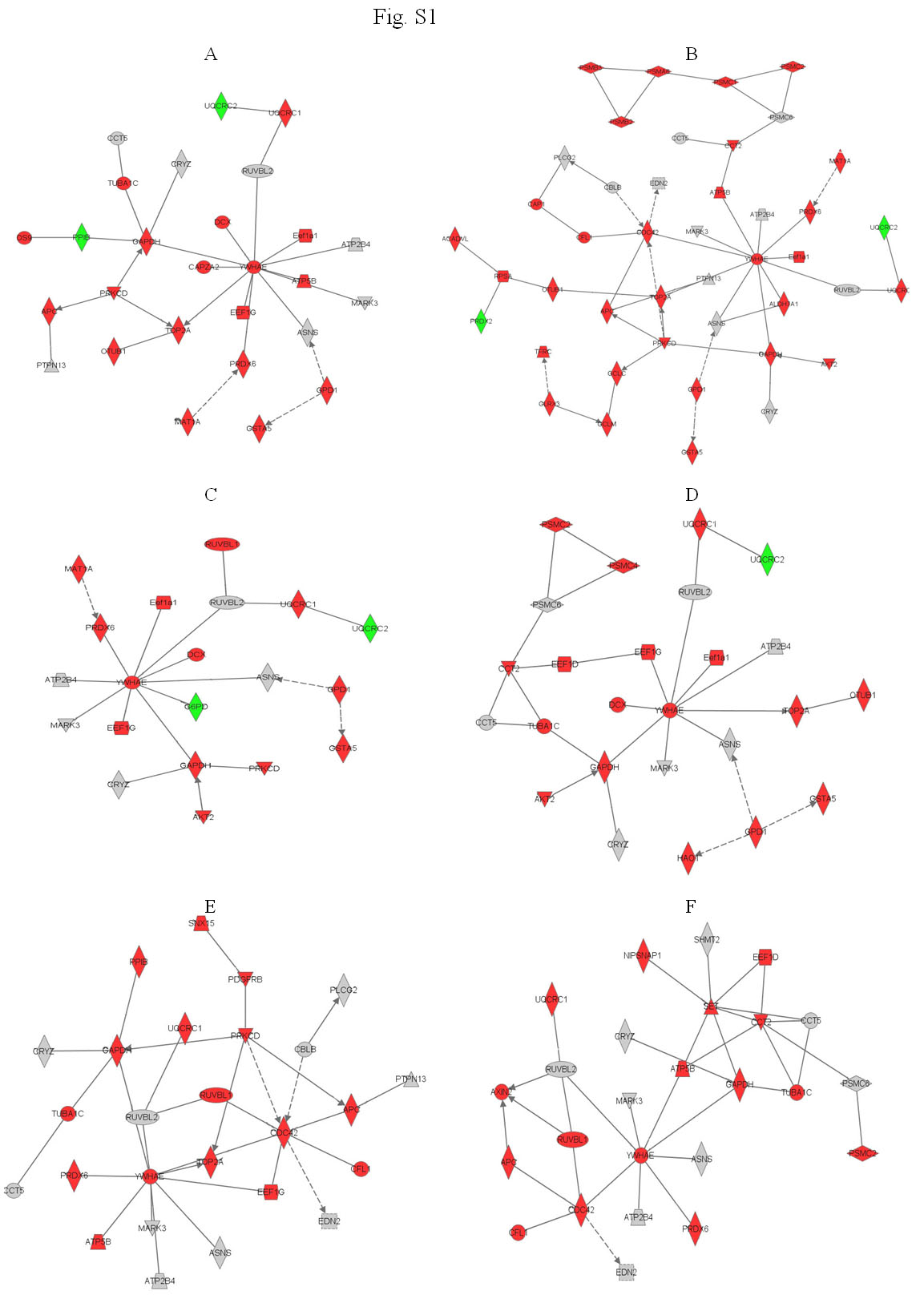

Supplement: Supplementary file 2 — Additional file 2: Figure S1: Pathway analysis results showing that differentially expressed proteins at each time points after 2/3 PH in rats can be sorted into a specific biological pathway. The root nodes in six subnetworks are all connected to YWHAE directly or indirectly. Red and green proteins were identified as differentially upregulated and downregulated, respectively. Other gray denoted the proteins that were down-regulated only in SO sample or detected only in SO sample. Lines connecting the molecules indicate molecular relationships. Real lines indicate direct interactions and dashed lines indicate indirect interactions. (JPEG 192 KB) [file 40659_2014_70_MOESM2_ESM.jpeg]
